# Altered behavioral and metabolic circadian rhythms in mice with disrupted NAD^+^ oscillation

**DOI:** 10.18632/aging.100368

**Published:** 2011-08-31

**Authors:** Saurabh Sahar, Veronica Nin, Maria Thereza Barbosa, Eduardo Nunes Chini, Paolo Sassone-Corsi

**Affiliations:** ^1^ Department of Pharmacology, University of California, Irvine, CA 92697, USA; ^2^ Department of Anesthesiology, Mayo Clinic College of Medicine, Rochester, MN 55902, USA; ^3^ Unite 904 of INSERM (Institut National de la Sante et de la Recherche Medicale) University of California, Irvine, CA 92697, USA

**Keywords:** Circadian Rhythm, Clock, NAD+, CD38, Amino acid metabolism

## Abstract

The Intracellular levels of nicotinamide adenine dinucleotide (NAD^+^) are rhythmic and controlled by the circadian clock. However, whether NAD^+^ oscillation in turn contributes to circadian physiology is not fully understood. To address this question we analyzed mice mutated for the NAD^+^ hydrolase CD38. We found that rhythmicity of NAD^+^ was altered in the CD38-deficient mice. The high, chronic levels of NAD^+^ results in several anomalies in circadian behavior and metabolism. CD38-null mice display a shortened period length of locomotor activity and alteration in the rest-activity rhythm. Several clock genes and, interestingly, genes involved in amino acid metabolism were deregulated in CD38-null livers. Metabolomic analysis identified alterations in the circadian levels of several amino acids, specifically tryptophan levels were reduced in the CD38-null mice at a circadian time paralleling with elevated NAD^+^ levels. Thus, CD38 contributes to behavioral and metabolic circadian rhythms and altered NAD^+^ levels influence the circadian clock.

## INTRODUCTION

Circadian rhythms occur with a periodicity of about 24 hours and regulate a wide array of metabolic and physiologic functions. A robust circadian clock allows organisms to anticipate environmental changes and to adapt their behavior and physiology to the appropriate time of day. Disturbances in the functionality of this “body clock” have been shown to lead to various diseases, such as sleep disorders, depression, metabolic syndrome and cancer [1,2]. Circadian rhythms are regulated by transcriptional and post-translational feedback loops generated by a set of interplaying clock proteins. The transcription factors CLOCK and BMAL1 operate as the master regulators of the clock machinery. CLOCK:BMAL1 heterodimers bind to promoters of clock controlled genes (CCGs) and regulate their expression. Some CCGs are special in the sense that they encode other core-clock regulators, such as *Period* and *Cryptochrome* genes, which negatively feedback on the clock machinery [1-3]. Recently, the deacetylase sirtuin 1 (SIRT1) was identified as a modulator of the circadian clock machinery that counterbalances the acetyltransferase activity of CLOCK [4-7]. Moreover, an additional novel transcriptional/enzymatic feedback loop that regulates the circadian clock has recently been uncovered [8,9]. The circadian clock controls the levels of NAD^+^ by regulating the expression of nicotinamide phosphoribosyltransferase (NAMPT), the rate-limiting enzyme in the salvage pathway of NAD^+^ biosynthesis [8,9]. NAD^+^ is also synthesized from the amino acid tryptophan by the *de novo* synthesis pathway. Tryptophan levels have been shown to oscillate in plasma [10], perhaps contributing to the oscillations in NAD^+^ levels. NAD^+^ is a cofactor and /or substrate for over 300 enzymes and acts as a cellular energy currency. SIRT1 is one such enzyme whose deacetylase activity is NAD^+^-dependent [11]. In addition, poly(ADP-ribose) polymerase (PARP)-1 and PARP-2 are NAD^+^-consuming enzymes, and their deletion raises NAD^+^ levels in mice [12,13]. Moreover, the SIRT1 and PARPs systems have been linked as they possibly use the same NAD^+^ cellular pool [14]. Circadian oscillations in NAD^+^ levels drive SIRT1 rhythmic activity [4]. SIRT1, in turn, is recruited to the *Nampt* promoter along with CLOCK and BMAL1. Thus, the circadian machinery is regulated by an enzymatic/transcriptional feedback loop, wherein SIRT1 regulates the levels of its own coenzyme [8,9]. Interestingly, PARP-1 activity has also been shown to display circadian oscillation [15]. These findings highlight the intimate connections between the circadian clock and cellular metabolism [16].

Since the clock machinery controls the cyclic levels of NAD^+^, we wondered about the importance of this oscillation with respect to circadian function. We thereby questioned whether an imbalance of NAD^+^ levels in animals with an intact clock system would lead to defects in circadian rhythms. To test this hypothesis, we analyzed the circadian behavior and metabolism of CD38-deficient (KO) mice which display very high levels of NAD^+^ in tissues such as the brain and liver [17,18]. CD38 is a membrane protein that has multiple enzymatic activities [19], the major being the hydrolysis of NAD^+^, through which it controls cellular NAD^+^ levels [20,21]. CD38 is also present on the inner nuclear membrane and regulates SIRT1 activity through modulation of NAD^+^ levels [17,22]. SIRT1 activity is higher in the CD38-KO mice [22]. Interestingly, CD38-KO mice are resistant to high-fat diet-induced obesity, in part through the activation of the SIRT1-PGC1α axis [23]. Our present study shows that chronically elevated levels of NAD^+^ in CD38-null mice lead to modulation of the circadian clock illustrated by altered circadian behavior, clock gene expression and amino acid metabolism.

## RESULTS

**Figure 1:**
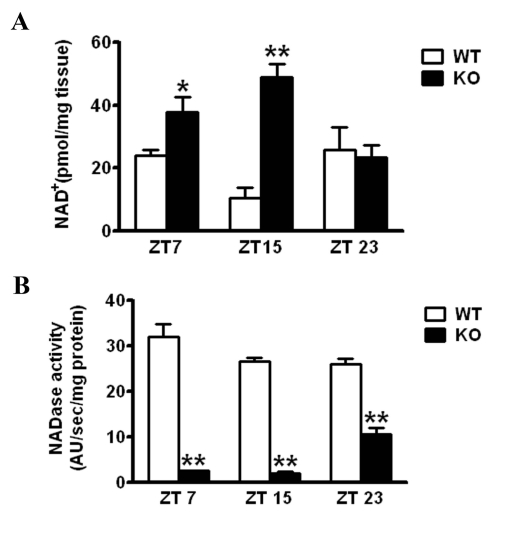
Effect of CD38 on NAD^+^ levels WT and CD38 KO mice entrained in 12 hr Light - 12 hr Dark (LD) cycles were sacrificed at indicated times and their liver was dissected out. **(A)** NAD^+^ concentration was measured by a cycling enzymatic assay. *, p<0.05 (WT vs KO ZT7); **, p<0.001 (WT vs KO ZT 15) [n=3 each time point] **(B)** NADase activity was measured by a flurometric assay. **, p<0.001 (WT vs KO for each time point) [n=3 each time point].

**Altered circadian NAD^+^ levels in CD38-null mice**. It has been reported that the CD38-KO mice have elevated levels of NAD^+^ in most tissues [17,18]. We analyzed the profile of NAD^+^ levels throughout the circadian cycle. NAD^+^ levels were measured at various zeitgeber times (ZTs) and were found to oscillate in the liver as described [9] (Fig. 1A). The oscillation of NAD^+^ was significantly altered in the liver of CD38-KO mice, being much higher than in WT mice at ZT7 and ZT15, with unchanged levels at ZT23 (Fig. 1A). Most remarkably, NAD^+^ levels in the CD38-KO mice were about 5 times higher than in WT animals at ZT15. Next, we wanted to determine whether the differences in NAD^+^ levels between WT and CD38-KO mice may be due to a change in NADase activity. Indeed, NADase activity in the liver of CD38-KO mice was drastically lower as compared to the WT animals at ZT7 and ZT15 (Fig 1B; ref. 13). Surprisingly, CD38-KO mice display high level of NADase activity (~50% of the WT activity) at ZT23. While the reasons for this could be due to a time-specific increase in the expression/activity of another unrecognized NAD^+^ glycohydrolase, this result explains the comparable NAD^+^ levels at ZT23.

**Figure 2:**
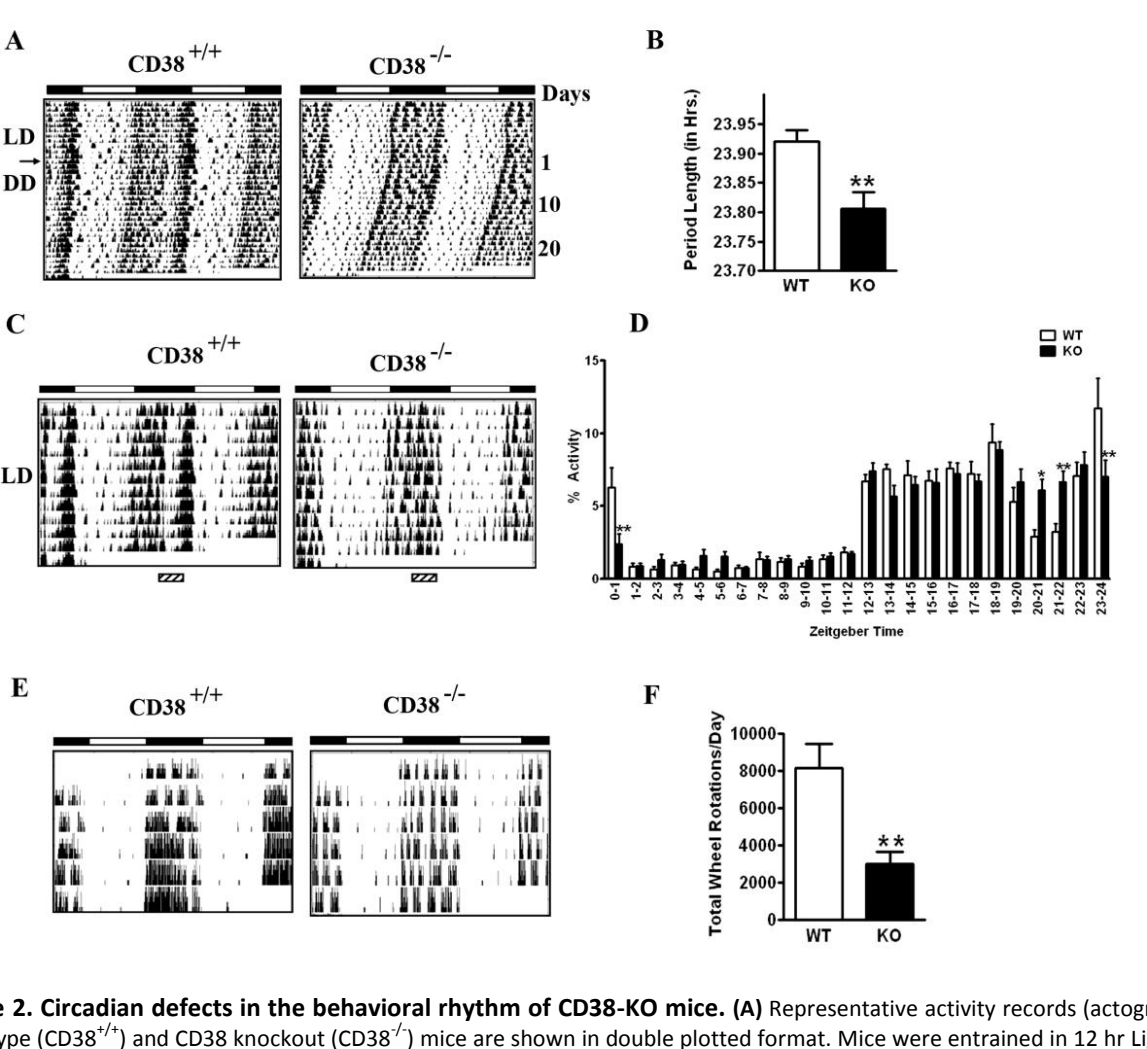
Circadian defects in the behavioral rhythm of CD38-KO mice (A) Representative activity records (actograms) of Wild type (CD38^+/+^) and CD38 knockout (CD38^-/-^) mice are shown in double plotted format. Mice were entrained in 12 hr Light - 12 hr Dark cycles (LD) and then placed in constant darkness (DD) from the light off (ZT12), on day 1. **(B)** Bar graph representing the period length of WT and CD38-KO mice. Measurement of the free-running period was based on the onset of activity in DD. Data is represented as mean ± S.E. * *, p = 0.008, n= 6, 8. **(C)** Representative actograms of Wild type (CD38^+/+^) and CD38 knockout (CD38^-/-^) mice in LD cycle. **(D)** Bar graph representing % daily locomotor activity in a one-hour period at the indicated ZTs. Data represents mean ± S.E of 10 days of activity. *, p < 0.05; **, p<0.01 compared to the corresponding wild type, n= 6, 8. **(E)** Representative actograms from wheel running activity of Wild type (CD38^+/+^) and CD38 knockout (CD38^-/-^) mice in LD cycle. **(F)** Bar-graph representing total number of wheel rotations per day. Data is represented as mean ± S.E. **, p = 0.008, n= 6, 5.

**CD38-KO mice display a shorter period length of locomotor activity**. The deregulation of NAD^+^ oscillation in the CD38-KO mice prompted us to monitor the circadian behavior of these mice. We analyzed the period length (*tau)* of locomotor activity under constant conditions. After entrainment on a 12 hour Light: 12 hour Dark (LD) cycle for more than 3 weeks, CD38 WT and KO mice were transferred to constant darkness (DD, free running) starting at the time of lights “off”, (ZT 12). This day was defined as day 1. We used passive (pyroelectric) infrared sensors to measure period length (*tau*) [24]. While the WT mice displayed a *tau* of 23.92 hours, the CD38-KO mice displayed a *tau* of 23.80 hours (Fig 2A, B). While this is a relatively small difference in *tau* (about 7 minutes), it is highly significant (P = 0.008, n=6, 8).

To establish whether WT and CD38-KO mice differed in the resetting responses to light, mice entrained to a LD cycle for several weeks were exposed to an 8 hr extension of the light period, followed by constant darkness. A phase delay is expected when mice are exposed to light during this period of night (ZT 12-20). Both WT and CD38-KO mice showed a similar phase angle of ~ 4 hr; however, the CD38-null mice displayed a further shortening of the period length to 23.75 hours, compared to 23.94 hours displayed by WT mice after the phase extension (Fig S1 A,B). These results indicate that the CD38-deficient animals have normal resetting responses to light, while their *tau* is shorter.

**Altered rest/activity rhythms in the CD38-null mice**. While monitoring locomotor activity of the WT and CD38-KO mice, we noticed a marked difference in their rest/activity patterns. The WT mice displayed a significant break from activity during the middle of the night; however, CD38-null mice did not appear to take this break (Fig. 2C, area over the striped bar). Instead, CD38-KO mice appeared to take multiple breaks, randomly spread throughout the day. Quantitation of this difference in activity revealed that while WT mice decrease their locomotor activity significantly between ZT20 and ZT22, CD38-null mice maintained almost constant levels of activity throughout the night (Fig. 2D). Significant differences in activity levels were also detected at several times during the circadian cycle. The differential activity pattern persists even under free running conditions (compare the actograms in Fig. 2A).

Analysis of the actograms indicated that the total locomotor activity in the CD38-KO mice might be lower than their WT counterparts. To accurately quantitate total locomotor activity, wheel-running activity was monitored. The actograms from the wheel-running analysis demonstrated that the CD38-KO mice have reduced locomotor activity (Fig. 2E). Total locomotor activity is reduced by ~3 fold in the CD38-KO mice, compared to the WT mice (Fig. 2F). The alterations in the rest/activity rhythms observed by the passive infrared sensors were also detected in the wheel-running activity. While WT mice took a break from wheel running only during a small period at late night, the CD38-KO mice took intermittent breaks. These results demonstrate that the rest/activity rhythm is disturbed in the CD38-deficient mice.

**Figure 3:**
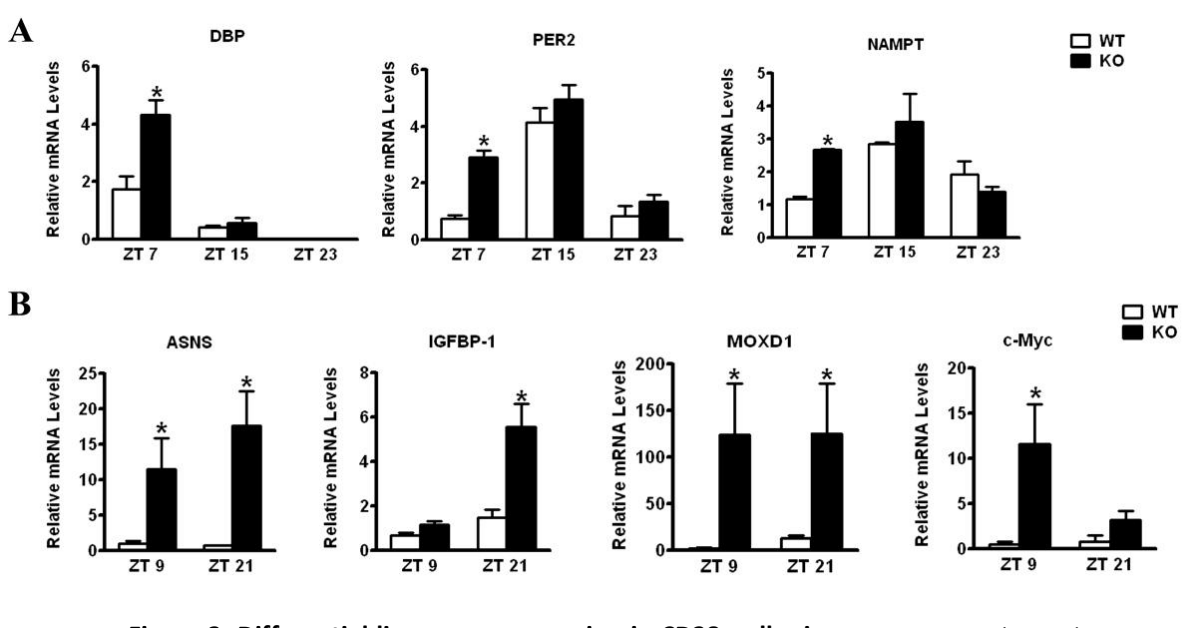
Differential liver gene expression in CD38-null mice Mice entrained in 12 hr Light - 12 hr Dark cycles were sacrificed at indicated times and their liver was dissected out.**(A)** RNA was prepared at indicated times, reverse transcribed, and real-time PCR was performed using primers for *Dbp*, *Per2*, *Nampt* and 18S rRNA. Data is represented as relative levels of indicated gene normalized to 18S rRNA.**(B)** Same as in (A), except real-time PCR was performed using primers for *Asns*, *Igfbp1*, *Moxd1* and c *-Myc*. Data is represented as relative levels of indicated gene normalized to 18S rRNA. *, p<0.05 compared to the corresponding wild type, n= 3 each time point.

**Ablation of CD38 results in disturbances in the peripheral clockwork**. The behavioral analysis of the CD38-KO mice indicates that deregulated and elevated levels of NAD^+^ alter central clock functions. To address whether peripheral clock function is also affected by deregulated NAD^+^ levels, we analyzed the circadian gene expression of representative clock genes from liver. WT and CD38-KO mice were entrained to a 12 hr LD cycle and livers were harvested at various circadian times. As shown in Fig. 3A, the amplitude of circadian gene expression of *Dbp,Per2* and *Nampt* is significantly higher in CD38-null than in WT mice, with most divergent levels at ZT7. These results indicate that peripheral clock is also affected by elevated NAD^+^ levels.

To establish if circadian metabolic pathways are influenced by deregulation of NAD^+^ in the CD38-KO mice, we performed a microarray analysis. Entrained WT and CD38-KO mice were sacrificed at two circadian times (ZT9 and ZT 21), and liver mRNAs were used for microarray analysis. Among the genes that are differentially regulated between WT and CD38-KO mice (Table S1), we validated 4 genes which were upregulated in the mutant mice (Fig. 3B). These genes were *Asparagine Synthetase* (*Asns*);*Insulin-like growth factor binding protein 1* (*Igfbp1*); *c-Myc*; and *monooxygenase, DBH-like 1* (*Moxd1*). Among these genes, *c-Myc* and *Igfbp1* have been shown to display a circadian profile of expression [25,26]. While *Asns* and *Moxd1* were elevated at both ZTs in the CD38-KO mice, *c-Myc* and *Igfbp1* elevation is more pronounced at ZT9 and ZT 21, respectively (Fig. 3B). Interestingly, SIRT1 might be involved in the regulation of *Asns* expression. SIRT1 activity highly correlates with *Asns* expression levels: *Asns* levels are high in CD38-KO mice (where the SIRT1 activity is high); and, conversely, *Asns* expression levels are very low in SIRT1 liver-specific KO mice (Fig. S2A).

The expression of *Asns* and *Igfbp1* is known to be upregulated under conditions of amino acid deprivation [27-29]. We verified that the *Asns* promoter is responsive to amino acid starvation. Indeed, *Asns*-promoter driven luciferase activity was induced over six-fold after amino acid depletion (Fig. S2B). Since most amino acids display a circadian profile in mouse plasma [10], these results prompted us to evaluate whether the circadian regulation of amino acid metabolism might be dependent on the presence of CD38.

**Figure 4:**
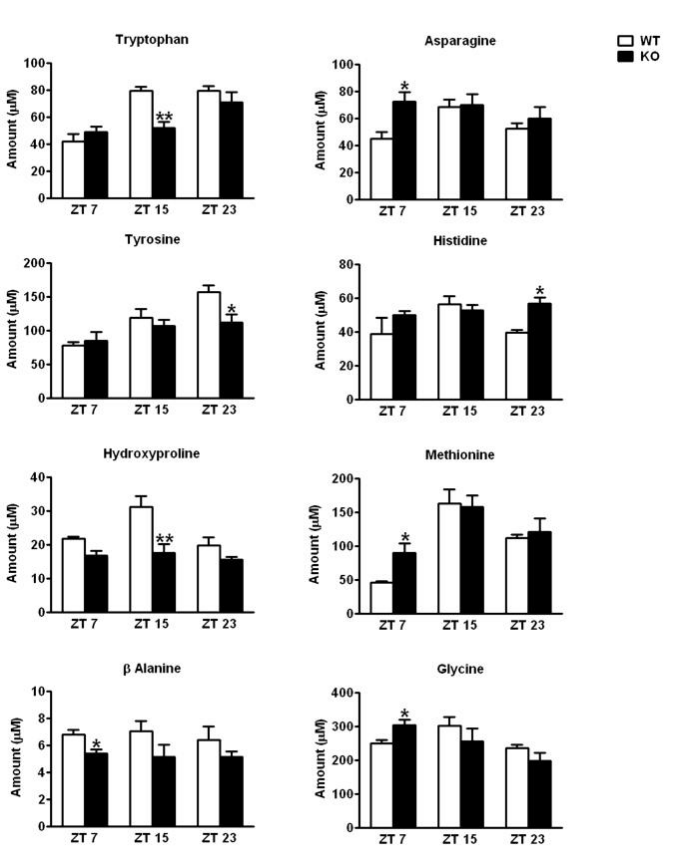
Alterations in the plasma amino acid levels in CD38-KO mice Mice Mice were entrained in 12 hr Light - 12 hr Dark cycles and blood was drawn at indicated times. Amino acid levels in plasma were determined as described in Materials and Methods. Amino acids that displayed statistically significant differences in abundance between the wild type and the CD38-KO mice are shown here. *, p<0.05; **, p<0.01 compared to the corresponding wild type, n= 3 each time point.

**Alterations in the plasma amino acid levels in the CD38-KO mice**. We performed a metabolomic study to determine the plasma amino acid levels in WT and CD38-KO mice along the circadian cycle, according to the method described by Lanza *et. al.* [30]. Most amino acid levels oscillated in a pattern consistent with that recently described by Minami *et. al.* [10]. We observed significant changes in the amino acid levels between the WT and the CD38-KO mice, at different circadian times. Four amino acids (Tryptophan, Hydroxyproline, Tyrosine, and β-Alanine) displayed significantly reduced levels at one or more circadian time in the plasma of CD38-KO mice (Fig. 4). On the other hand, four amino acids (Asparagine, Glycine, Histidine and Methionine) displayed higher levels in the CD38-KO mice (Fig. 4). These results demonstrate that the circadian oscillations in amino acids levels are compromised in the CD38 KO mice, thereby establishing a direct link between specific amino acid metabolic pathways and controlled NAD^+^ levels.

## DISCUSSION

Our current work has addressed the question whether alterations in NAD^+^ rhythmicity *in vivo* could affect the functioning of central and peripheral clocks. For this purpose we explored the circadian behavior of CD38-KO mice which have elevated NAD^+^ levels. We observed that oscillations in NAD^+^ levels were altered in the liver of CD38-KO mice. Strikingly, at ZT 15 (the trough of NAD^+^ levels in WT liver), NAD^+^ levels peaked in CD38-KO mice and were ~ 5 times higher than those in the liver of WT mice. Surprisingly, NAD^+^ levels were similar between WT and CD38-KO mice at ZT23. This could be due to an increase at this circadian time of NADase activity in CD38-null mice. Although CD38 is the major NAD^+^ hydrolase in cells, it is possible that in its absence other NAD^+^ hydrolases (such as CD157, a gene duplication product of CD38) could be induced as a compensatory mechanism.

CD38 also plays a role in the synthesis of second messengers cyclic ADP-Ribose (cADPR), ADPR, and nicotinic acid adenine dinucleotide phosphate (NAADP) [19]. CD38 can produce one molecule of cADPR for every 100 molecules of NAD^+^ hydrolyzed [21,31], suggesting that the major enzymatic activity of CD38 is the hydrolysis of NAD^+^. CD38 regulates SIRT1 through a cADPR independent pathway [23]. Acetylation of several SIRT1 targets, such as p53, is reduced in the CD38-KO mice [22,23], and CLOCK-induced BMAL1 acetylation is enhanced by ectopic expression of CD38, possibly through modulation of SIRT1 activity (Fig. S3). In keeping with this observation, several circadian behavioral defects were observed in the CD38-KO mice. Their free-running period length is ~ 7 minutes shorter than the WT animals. Although this difference may appear small, deletion of CLOCK, the master regulator of circadian rhythms, changes the period length by only 20 minutes [32]; and mutants in NPAS2 (a paralog of CLOCK highly expressed in the brain) show only a 12 minute shortening in period length [33]. Interestingly, CD38-null mice displayed an altered pattern of rest/activity rhythm, reminiscent of that reported for NPAS2 mutant mice [33]. WT mice on a C57/BL6 background are known to take a break at late night (ZT 20-22), which generally corresponds to a short nap [33]. Both CD38-null mice and NPAS2 mutant mice remain active at that time. However, differently from NPAS2 mutant mice, CD38-deficient mice appear to keep taking intermittent breaks throughout the day. Moreover, the total locomotor activity of CD38-null mice is also significantly lower. This reduction in locomotor activity could be attributed to increased SIRT1 activity in the CD38-KO mice (17), since transgenic mice overexpressing SIRT1 display a similar reduction in locomotor activity [34]. The altered rest/activity rhythm in the CD38-KO mice does not seem to be caused by defects in the motivation for running. These mice took several breaks from running and kept running at regular intervals. This behavior could potentially be described as a fatigue syndrome. Further studies are required to prove this hypothesis.

The central circadian clock is located in the suprachiasmatic nucleus (SCN) of the hypothalamus, while peripheral clocks are present in most organs [35]. We have shown that ablation of CD38 results in elevated levels of NAD^+^ in the liver, leading to perturbation in clock function. The stringency of circadian oscillation is compromised in the absence of CD38, as demonstrated by the gene expression profiles of *Dbp,Per2* and *Nampt.* The deregulation of circadian gene expression extends to several genes involved in amino acid metabolism, which we found to be upregulated in the liver of CD38-deficient mice. These genes were *Asparagine synthetase* (*Asns*);*Insulin-like growth factor binding protein 1* (*Igfbp1*); *c-Myc*; and *monooxygenase, DBH-like 1* (*Moxd1*). The liver is a major organ involved in the amino acid metabolism. Nutritional stresses, such as reduced availability of an amino acid, can initiate a signaling cascade referred to as the amino acid response (AAR) pathway [29]. *Asns* and *Igfbp1* are genes that are known to be upregulated by the AAR pathway. Asparagine synthetase converts amino acids aspartate and glutamine into asparagine and glutamate. Interestingly, SIRT1 activity positively correlates with *Asns* expression levels. SIRT1 has also been shown to induce the expression of *Igfbp1* [36]. Low *Igfbp1* levels are considered to be markers of metabolic syndrome [37] and the *Igfbp1* transgenic mice are protected from diet-induced obesity [38], a trait shared by the CD38-null mice [23]. c-MYC, besides having the well characterized role in cell cycle regulation, also regulates glutaminolysis [39]. MOXD1 is a monoxygenase with a yet unidentified function; however, monooxygenses are known to regulate conversion of one amino acid into another (e.g. phenylalanine hydroxylase, a monooxygenase, converts phenylalanine to tyrosine). It is interesting to speculate that MOXD1 might also function in inter-conversion of amino acids and is upregulated under conditions of deficiency of a subset of amino acids.

A metabolomic analysis confirmed our hypothesis that circadian oscillations in amino acid levels are disrupted in the absence of CD38. Levels of tryptophan, tyrosine, hydroxyproline and β-alanine were found to be reduced in CD38-null mice, whereas asparagine, glycine, histidine and methionine were elevated. Moreover, oscillations in most of these amino acids were dampened in mutant mice. Changes in asparagine and tryptophan levels are of special interest. Since *Asns* levels are higher in CD38-null mice, we predicted that asparagine levels would also be higher. Surprisingly, asparagine levels were high only at ZT7, although *Asns* expression was constitutively higher in mutant mice. This suggests that ASNS activity might be modulated in a circadian manner. As tryptophan is a source for NAD^+^ biosynthesis, it is intriguing that levels of NAD^+^ were very high and levels of tryptophan were significantly reduced at ZT15 in CD38-null mice, alluding to the consumption of tryptophan during NAD^+^ biosynthesis. Finally, it can be concluded that the altered levels of some amino acids might trigger the amino acid response pathway in CD38-KO mice.

An intriguing speculation relates to the notion that amino acids and their metabolites can also function as neurotransmitters or their precursors. Glutamate, aspartate, glycine and γ-amino butyric acid (GABA) are neurotransmitters. Tryptophan is a precursor for serotonin, whereas tyrosine is a precursor for catecholamines, such as dopamine, epinephrine and norepinephrine. An imbalance in these amino acids can potentially change the concentration of certain neurotransmitters in the brain. Further studies will establish whether changes in these neurotransmitters are responsible for alterations in the rest/activity rhythms and locomotor activity in the CD38-null mice.

In conclusion, our results reveal a role for NAD^+^ homeostasis and SIRT1 activity in circadian regulation of behavioral and metabolic rhythms. Increased NAD^+^ levels and concomitant activation of SIRT1 might lead to reduction in several amino acids, which activates the AAR pathway in CD38-null mice. Our findings underscore the importance of circadian control in amino acid metabolism.

## MATERIALS AND METHODS

### Animals

Generation of CD38-deficient mice (C57BL/6J.129 *CD38*^−/−^, N12 backcross) has been described [40]. Liver-specific *Sirt1*^−/−^ mice have also been described [4]. Mice housed in individual cages were entrained on a L12:D12 (12 h light-12 h dark) cycle for two weeks before analyses. Mice were sacrificed at specified circadian times. All research involving vertebrate animals has been performed under protocol approved by the Institutional Animal Care and Use Committee (IACUC). Animals are monitored on a daily basis by both the lab and University Lab Animal Resources (ULAR) veterinary staff for signs of distress, pain, and/or infection, and are given *ad libitum* access to food and water. Cages were cleaned on a weekly basis and when visibly soiled to maintain a clean environment. All husbandry procedures and welfare policies are conducted according to the Guide for the Care and Use of Laboratory Animals, set forth by the Institute of Laboratory Animal Resources, Commission on Life Sciences, and National Research Council.

### NAD^+^ measurements

NAD^+^ was measured as described previously [17]. Briefly, frozen tissue was pulverized with a pestle and mortar in liquid nitrogen, immediately extracted in ice-cold 10% trichloroacetic acid (sigma) and sonicated with 3 pulses of 3 seconds. After a short centrifugation the supernatant was extracted with 2 volumes of a combination of 1,1,2-trichloro-1,2,2-trifluroethane : trioctylamine in a 3 to 1 ratio. The samples were vigorously vortexed and 2 phases were allowed to separate at room temperature. The pH of the extracted aqueous layer containing the NAD^+^ was adjusted with 1M Tris pH 8. The NAD^+^ concentration was measured by a cycling enzymatic assay. The samples were diluted in 100 mM NaH_2_PO_4_, pH 8 and incubated with a solution containing 0.76% ethanol; 4 μM flavine mononucleotide; 40 μg/ml alcohol dehydrogenase; 0.04 U/ml diaphorase; and 8 μM resazurin in buffer 100 mM NaH_2_PO_4_, pH 8. The fluorescence (excitation 544, emission 590) was monitored over time in a fluorometric plate reader (Spectramax Gemini XPS, Molecular devices). A NAD^+^ standard curve was performed using yeast NAD^+^ (sigma). NAD^+^ concentration was expressed as pmol per mg of tissue.

### NADase activity

The NADase activity in liver samples was measured as described previously [17]. Frozen liver samples were homogenized in sucrose 0.25M - Tris 40mM, pH 7.4 buffer supplemented with protease inhibitors (Roche), and centrifuged 10 minutes at 11200 g. 200 μg of homogenate was incubated with 100 μM of 1,N^6^-etheno-adenine dinucleotide (etheno-NAD)(Sigma) and the fluorescence was measured in a fluorometric plate reader (Spectramax Gemini XPS, Molecular devices). The change in fluorescence over time (excitation 300nm, emission 410) was followed and the NADase activity was expressed as the change in the arbitrary units of fluorescence per second per mg of protein.

### Analysis of behavioral rhythms

Circadian rhythms in locomotor activity was analyzed as described [24]. Briefly, locomotor activity was detected using running wheels and passive (pyroelectric) infrared sensors (PU-2201; EK Japan). Locomotion data were collected using the VitalView data acquisition system (Mini-Mitter) using a sampling interval of 5 min. Actograms were acquired using Actiview Biological Rhythm Analysis software (Mini-Mitter). Circadian period and phase shift of the activity rhythms were analyzed by Clocklab software (Actimetrics).

### Quantitative Real-Time RT-PCR

Each quantitative real-time RT-PCR was performed using the PTC-200 real time detection system (MJ Research). The PCR primers are available upon request. For a 20 μl PCR, 50 ng of cDNA template was mixed with the primers to final concentrations of 200 nM and 10 μl of iQ SYBR Green Supermix (BIO-RAD), respectively. The reaction was first incubated at 95^0^C for 3 min, followed by 40 cycles at 95^0^C for 30 s and 60^0^C for 1 min.

### Luciferase assays

Hepa1c1c7 cells (ATCC, Manassas, VA) were cultured in Minimal Essential Media supplemented with 10% FBS and antibiotics. Cells growing in 24-well plates were transfected with indicated plasmids using BioT transfection reagent (Bioland Scientific LLC, Cerritos, CA) according to manufacturer's recommendations. Plasmid expressing 3.4 kb ASNS-promoter-driven luciferase was a gift from Dr. Pierre Fafournoux [41]. The total amount of DNA applied per well was adjusted by adding an empty vector. Cell extracts were subjected to luminometry-based-luciferase assay (Promega), and luciferase activity was normalized by β-galactosidase activity. All experiments were performed in multiple replicates.

### Amino acid analysis

Amino acid concentrations in plasma samples were determined at the Metabolomics facility at Mayo Clinic College of Medicine, Rochester, Minnesota according to the method described by Lanza *et. al.* [30]. Briefly, plasma samples were deproteinized with cold MeOH prior to derivatization using 6-aminoquinolyl-N-hydroxysuccinimidyl carbamate. Amino acid levels were then determined by LC-MS/MS.

### Data Analyses

Results are expressed as means ± SE of multiple experiments.Student *t* tests were used to compare 2 groups or ANOVA with the Bonferroni post tests for multiple groups using Prism software (Graph Pad). Statistical significance was detected at the 0.05 level.

## SUPPLEMENTAL DATA


