# Supplementary material for: Altered behavioral and metabolic circadian rhythms in mice with disrupted NAD+ oscillation
Source: Aging (Albany NY). 2011 Aug 31;3(8):794–802. doi: 10.18632/aging.100368 (PMC3184980; doi:10.18632/aging.100368)
Supplement: Supplementary file 1 [file aging-03-794-s001.pdf]

SUPPLEMENTARY FIGURE LEGENDS:

Fig S1: Mice were entrained in 12 hr Light – 12 hr Dark cycles (LD) and moved to constant darkness following 8 h of light prolongation on the last day of LD cycle. Locomotor activities were monitored by infrared sensors. **(A)** Representative actograms from locomotor activity of Wild type ($CD38^{+/+}$) and CD38 knockout ($CD38^{-/-}$) mice. Arrow indicates the day of transition to DD. **(B)** Bar graph representing the period length of WT and CD38-KO mice. Measurement of the free-running period was based on the onset of activity in DD. Data is represented as mean ± S.E. **, p = 0.006, n= 6, 6.

Fig S2: **(A)** Mice entrained in 12 hr Light – 12 hr Dark cycles were sacrificed at indicated times and their liver was dissected out. RNA was prepared at indicated times, reverse transcribed, and real-time PCR was performed using primers for *Asns* and 18S rRNA. Data is represented as relative levels of indicated gene normalized to 18S rRNA. *, $p<0.05$, n = 4. **(B)** Hepa1c1c7 cells were transfected with a luciferase reporter under the control of 3.4Kb ASNS promoter. 6 hours post-transfection, medium was changed to either complete medium or medium depleted in methionine, cystine and glutamine. 18 hours later cells were harvested and luciferase activity was measured. Luciferase activity was normalized to β-galactosidase activity. ***, p = 0.00008, n = 4.

Fig S3: HEK 293 cells were transfected with indicated plasmids and 48 hours post-transfection total lysates were prepared. BMAL1 was immunoprecipitated by anti-flag antibody and resolved by SDS-PAGE. Acetylated BMAL1 levels were detected by anti-acetyl BMAL1 antibody. Total BMAL1 and CLOCK levels were detected by anti-Myc antibody. Nicotinamide treatment was used as positive control.

SUPPLEMENTARY TABLE LEGEND:

Table S1: Genes found to be differentially expressed in the WT and CD38-KO livers at the indicated circadian times.

Fig. S1

A

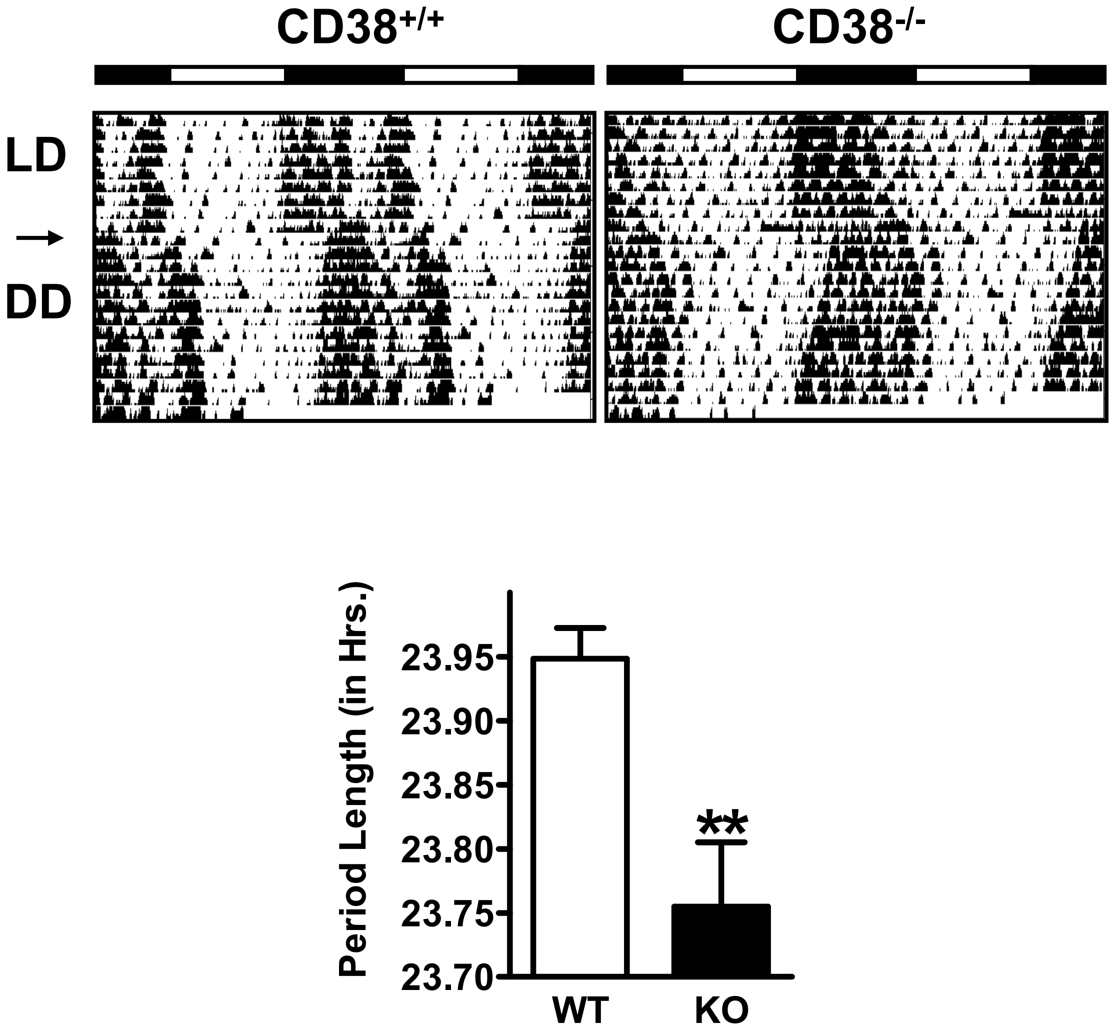
CD38+/+
CD38-/-
LD
DD
B
Period Length (in Hrs.)
23.95
23.90
23.85
23.80
23.75
23.70
**
WT
KO

Fig. S2

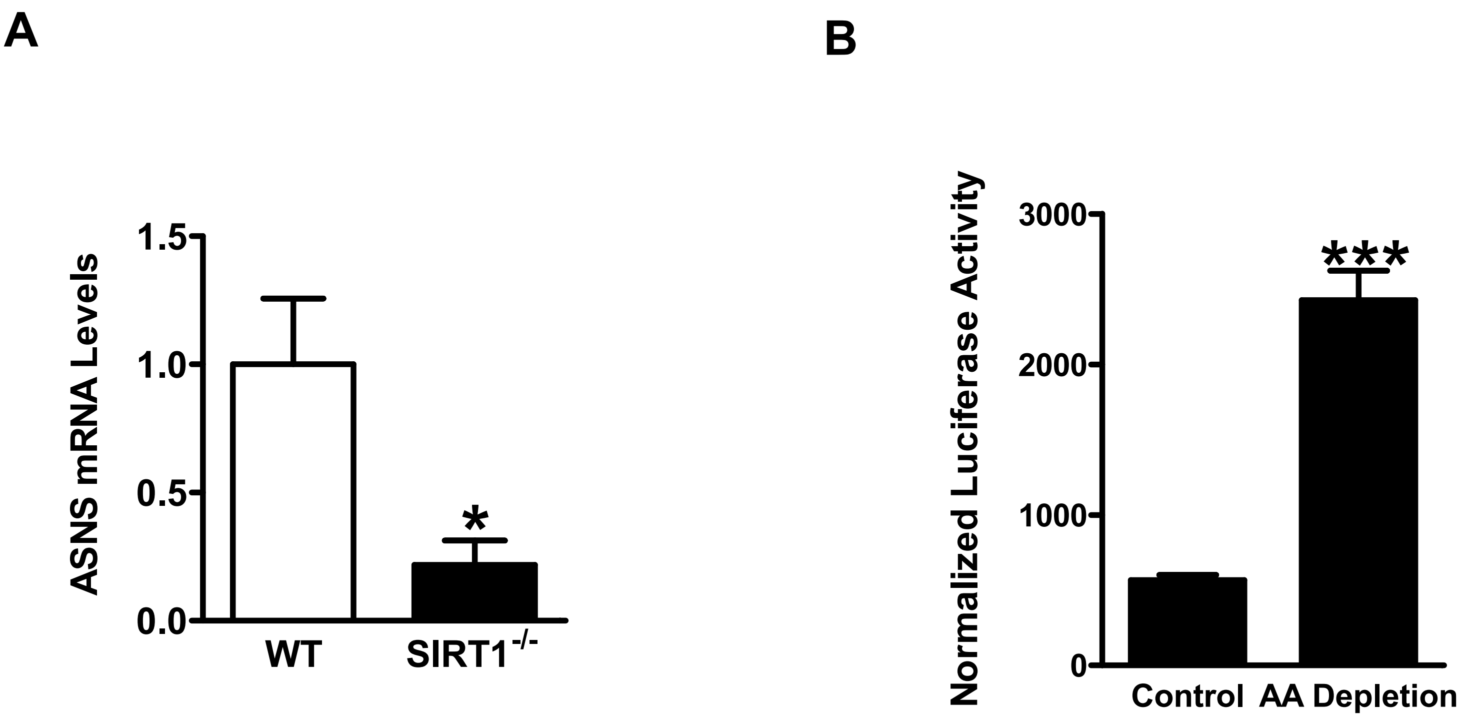
A
ASNS mRNA Levels
1.5
1.0
0.5
0.0
*
WT
SIRT1-/-
B
Normalized Luciferase Activity
3000
2000
1000
0
***
Control
AA Depletion

Fig. S3

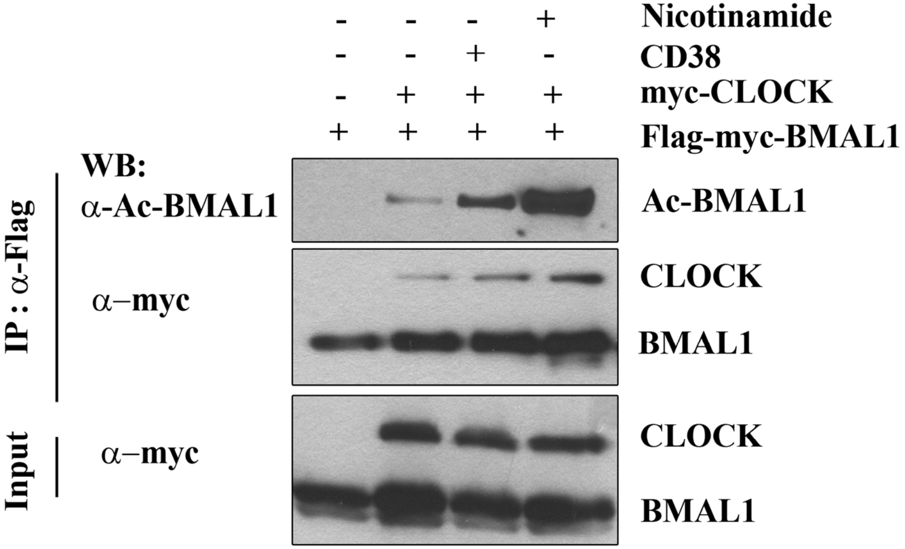

- - - + Nicotinamide
- - + - CD38
- + + + myc-CLOCK
+ + + + Flag-myc-BMAL1
WB:
α-Ac-BMAL1
Ac-BMAL1
IP : α-Flag
α–myc
CLOCK
BMAL1
Input
α–myc
CLOCK
BMAL1

Table S1

Gene	Description	WT		CD38$^{-/-}$	
		ZT9	ZT21	ZT9	ZT21
Acot3	acyl-CoA thioesterase 3	203.5166	469.7717	588.9106	203.797
Asns	asparagine synthetase	475.0676	212.9754	4012.233	1514.044
Cish	cytokine inducible SH2-containing protein	171.4409	622.628	1733.328	405.2249
Gadd45g	growth arrest and DNA-damage-inducible 45 gamma	95.73499	246.5598	490.1747	199.6584
Socs2	suppressor of cytokine signaling 2	326.755	1301.482	735.9031	1069.586
Gdpd3	glycerophosphodiester phosphodiesterase domain containing 3	137.4398	148.2955	4980.955	258.5633
Moxd1	monooxygenase, DBH-like 1	189.4579	198.0222	2175.187	449.5633
Myc	myelocytomatosis oncogene	167.424	321.2134	919.8262	297.3
Kcnt2	potassium channel, subfamily T, member 2	62.46495	96.45612	318.9425	139.1038
Igfbp1	insulin-like growth factor binding protein 1	298.5939	316.882	231.6723	605.2462
Lcn2	lipocalin 2	674.6613	723.3514	2013.737	910.7525
Cxcl13	chemokine (C-X-C motif) ligand 13	570.1917	527.0361	165.1674	751.9225
